# Behavior of *Cucumis sativus* L. in presence of aluminum stress: Germination, plant growth, and antioxidant enzymes

**DOI:** 10.1002/fsn3.2294

**Published:** 2021-05-05

**Authors:** Rim Kouki, Rim Ayachi, Renata Ferreira, Noomene Sleimi

**Affiliations:** ^1^ LR. RME‐Resources, Materials and Ecosystems Faculty of Sciences of Bizerte University of Carthage Bizerte Tunisia; ^2^ CERENA Centro de Recursos Naturais e Ambiente Instituto Superior Técnico Universidade de Lisboa Lisboa Portugal

**Keywords:** Al accumulation, antioxidant enzymes, *Cucumis sativus* L., germination parameters, growth

## Abstract

Aluminum (Al) is an ubiquitously present element in soil; it is considered as a major problem in crop production that affects plant growth and development on acid soils. The aim of this work was to determine the germination parameters, to quantify the water status and growth, to assess the Al accumulation, and antioxidant enzyme activities in plants to evaluate the stress exerted by aluminum in *Cucumis sativus* L. For germination test, increasing doses of Al were used (0, 200, 500, 1,000, and 2,000 μM). Results showed that germination was stimulated with 500 of Al. Aluminum effects on development were studied by treating the plants with different concentrations of Al (100, 200, 300, and 500 µM, Al) during 45 days. As regards to the plant's growth, water content, and dry biomass production there was a slight increase. On the other hand, the activities of the antioxidant enzymes were disturbed by aluminum stress. Data indicate that the catalase (CAT) activity showed a decrease in the different parts of the plant. However, guaiacol peroxidase (GPX) and ascorbate peroxidase (APX) activities were significantly stimulated. Studying the effects of Al‐induced stress allowed us to conclude that cucumber has a high ability to accumulate this element in the roots.

## Graphical abstract

The treatment of cucumber seeds with Al improves the germination percentage. Al stimulates the production of biomass and the water content in plants. Al disturbs the activities of antioxidant enzymes CAT, APX, and GPX in plants. In cucumber, the accumulation of Al occurs mainly in roots.

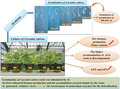

## 1 INTRODUCTION

Aluminum (Al) toxicity has emerged as a major limitation to agronomic performance in acidic soils. In fact, Al is ubiquitously distributed as the third most abundant element in the Earth's crust in different forms in soil environments and represents approximately 7%–8% of its mass (Bojórquez‐Quintal et al., 2017). A significant correlation has been found between pH soil and the phytotoxicity by Al species. Hence, the multiple forms of Al, their concentrations, speciation, and toxicity in the soil environment depend on pH level and the chemistry of the soil solution (Kisnieriené & Lapeikaité, 2015).

The trivalent Al^3+^ is the most abundant form and very toxic; it has also the greatest impact on plant growth (Rahman et al., 2018). In acid soils, Al toxicity is one of the main factors limiting crop production by reducing seed germination in several plant species, mainly because of hormonal imbalance (Roshani et al., 2014). In fact, seed germination and seedling development are the most sensitive physiological stages in plants, especially in the presence of metals, since the defense processes are affected, being often regarded as an important index to evaluate plant tolerance to heavy metals (Talebi et al., 2014).

Aluminum is not an essential element for growth. In soil, beyond the threshold concentration, Al can induce toxic effect on plants, leading to the hindrance of plant root growth and reduced nutrition and water availability (Fan et al., 2020) where the physiological disturbances intensity depends on the level of accumulation of the metal ion within the plant tissue (Chibuike & Obiora, 2014). In fact, root growth was significantly inhibited by toxic Al ions in acid soil (Pereira, 2018) which is manifested by disordered arrangement of cells, with deformed shapes and altered structure, and a reduced meristematic zone of the root tips (Wang et al., 2016).

Otherwise, the phytotoxic Al leads to an excessive generation of reactive oxygen species (ROS) that can mediate antioxidant defense mechanisms, such as hydrogen peroxide and singlet oxygen, as those ROS were detected for example in the leaves of *Oryza sativa* (Kuo & Kao, 2003). Among the defense mechanisms, we can mention the enzymatic antioxidant system. Excessive doses of metallic trace elements (TME) may result in a decrease or an increase of the activity of antioxidant enzymes such as catalase (CAT) guaiacol peroxidase (GPX) and ascorbate peroxidase (APX) which play a crucial role in the neutralization of ROS (Zheng et al., 2010).

Taking into account the considerations above, the experiments carried out in this work aim to better understand and to evaluate the mechanisms involved in tolerance of stress exerted by Al by the investigation of the Al toxicological effects on seed germination, and plant's growth in cucumber (*Cucumis sativus*). Moreover, an assessment of Al accumulation in the tissues and the antioxidant enzyme activities of CAT, GPX, and APX were required for a better evaluation of Al potential toxicity.

## 2 MATERIAL AND METHODS

### 2.1 Plant material and culture

The seeds of *Cucumis sativus* were soaked for 2 hr in distilled water to ensure the lift of dormancy. Germination was carried out in Petri dishes with a double layer of filter paper fully moistened up with the test solutions made at different Al concentrations: 0, 200, 500, 1,000, and 2,000 μM. We used 8 Petri dishes per treatment and each one contained 10 seeds.

The experiment was conducted in a growth chamber at 25°C during a period of 12 days, with a periodic watering by treatment solutions in order to maintain the seeds imbibition. In fact, the germination was followed after 24 hr of sowing with a daily count of germinated seeds (every 2 hr).

Concerning the cucumber crops, the plants were grown on an inert substrate (1:2 (v/v) mixture of gravel and perlite) in a greenhouse under semi‐controlled conditions with a natural photoperiod, with temperatures ranging from 15 to 25°C (night‐day), and relative humidity between 60% and 90%. The seedling was irrigated (3 times a week) with the nutritive solution of Hewitt (1966) enriched with iron as complex EDTA‐K‐Fe and micronutrients as mixture of salts (MnCl_2_; CuSO_4,_ 5H_2_O; ZnSO_4_, 7H_2_O; (NH_4_)_6_Mo_7_O_24_, 4H_2_O; and H_3_BO_3_). The solution‐pH varied between 6.6 and 7.1. After 30 days of sowing, plants were divided into 5 groups (10 plants for each group grown separately in pots) and treated during 45 days with different doses of Al (0 (control), 100, 200, 300, and 500 µM) that were added to the nutrient solution.

On the harvest day, as a first step, a cold solution of CaCl_2_ was used to eliminate trace elements adsorbed into roots (Stolt et al., 2003) and then rinsed with cold distilled water. Plants were separated into roots and shoots.

The obtained plant material was split in two parts: one part was frozen in liquid nitrogen and kept at −80°C, the other part was dried in an oven at 70°C for 10 days, and finally conditioned according to the analyzes to be carried out. The determination of the fresh weight (FW) and the dry weight (DW) was carried out before and after drying as well as the water content (WC), which was determined as follows:

$$\text{WC}=\left(\text{FW}‐\text{DW}\right)/{\text{DW and expressed in mL of H}}_{2}{\text{O g}}^{‐1}\;\text{DW}.$$

### 2.2 Germination parameters

- The germination percentage (GP) was calculated by relating the number of seeds germinated to the total number of seeds tested (Ashraf & Abu‐Shakra, 1978).

GP=100∗(the number of seeds germinated/total number of seeds)

- Germination capacity (GCp) is the percentage of seeds that have been germinated during the germination process (Labouriau, 1983) and it was tested by the formula:

GCp=ni/N

Where ni is the cumulative number of seeds germinated at each observation. N is the total number of seeds that is set to germinate.

- The time (T_50_) corresponds to 50% of the germination, and it is expressed as indicated in the formula (Salehzade et al., 2009):

$T_{50}=\mathrm{ti}+\left(\frac{\left(N/2‐\;\mathrm{ni})(\mathrm{tj}‐\mathrm{ti}\right)}{\mathrm{nj}‐\;\mathrm{ni}}\right)$.

With N: the final number of seeds sprouted. ni_50_, nj_50_: the number of accumulated seeds corresponding to the time when ni < N/2 < nj. ti, tj: the time corresponding to ni and nj.

- The germination velocity coefficient (GVC) is the reciprocal of the mean germination time (Ranal & Garcia de Santana, 2006):

$\mathrm{GVC}=\left(\frac{100(n1+n2+\cdots ..\mathrm{nx})}{n1t1+n2t2+\cdots .\mathrm{nxtx}}\right)$.

With nx: the number of seeds sprouted for an observation x. tx: the day corresponding to the germination of the seeds.

- The germination index (GI) was calculated as described in the Association of Official Seed Analysts (AOSA, 1991) according to the formula:

$$\mathrm{GI}=\frac{\mathrm{nb}\;\mathrm{of}\;\mathrm{sprouted}\;\mathrm{seeds}}{\;\mathrm{the}\;\mathrm{first}\;\mathrm{day}\;\mathrm{of}\;\mathrm{counting}\;}+\cdots +\frac{\mathrm{nb}\;\mathrm{of}\;\mathrm{sprouted}\;\mathrm{seeds}}{\;\mathrm{the}\;\mathrm{last}\;\mathrm{day}\;\mathrm{of}\;\mathrm{counting}}$$

### 2.3 Dosage of Al accumulation

Dry plant material was digested by mixture of the 3 acids (HNO_3_/H_2_SO_4_/HClO_4_; at the volume proportion 10:1:0.5) (Sghaier et al., 2019). The mineralization was conducted during 2 hr at 110°C. Then, the extracts samples were diluted by the nitric acid 0.5% and filtered. The Al content in plant tissues was determined by atomic absorption spectrometry (Perkin Elmer PinAAcle 900T, USA) at *λ* = 309.27 nm in N_2_O/C_2_H_2_ flame.

### 2.4 Enzymatic assays

Protein extraction was carried out grinding 400 mg of fresh plant material in 2 ml of extraction buffer (50 Mm KH_2_PO_4_/K_2_HPO_4_, pH 7.0; 5 mM Na‐ascorbate and 0.2 mM EDTA). After that, a filtration was carried out through four layers of miracloth, and then, the homogenate was centrifuged at 4,830 *g* for 15 min at 4°C. The obtained supernatant was used to measure the activity of the antioxidant enzymes (CAT, APX, and APX).

The CAT activity was determined according to Asada (1999) at 240 nm by the decrease of the optical density of a reaction mixture containing 50 μl of the crude enzymatic extract, 50 mM H_2_O_2_, and 25 mM potassium phosphate buffer (pH 7).

The spectrophotometric assay of GPX activity was performed as described by Fielding and Hall (1978). The reaction mixture contained 10 µl of the crude enzyme extract, 30 mM H_2_O_2_, 25 Mm phosphate buffer (pH 7), and 9 mM guaiacol.

The measurement of the APX activity was carried out according to Nakano and Asada (1981). The reaction is followed by measuring ascorbate consumption at 290 nm in the reactionnal mixture containing 40 μl of the enzymatic extract, 2 mM H_2_O_2_, 25 mM potassium phosphate buffer (pH 7), 0.5 mM sodium ascorbate, and 0.1 mM EDTA.

### 2.5 Statistical analysis

All samples were analyzed for at least five replicates and mean values and standard deviation (±) are presented in bars in figures. The effects of TME on the variability of the studied parameters were evaluated using single‐factor analysis of variance (ANOVA1) by STATISTICA software to determine if a given factor has a significant effect. For the comparison of the means, the Tukey HDS test was used which gives the significant differences of these data at *p* < .05 and at *p* < .01.

## 3 RESULTS

### 3.1 Germination parameters

**FIGURE 1:**
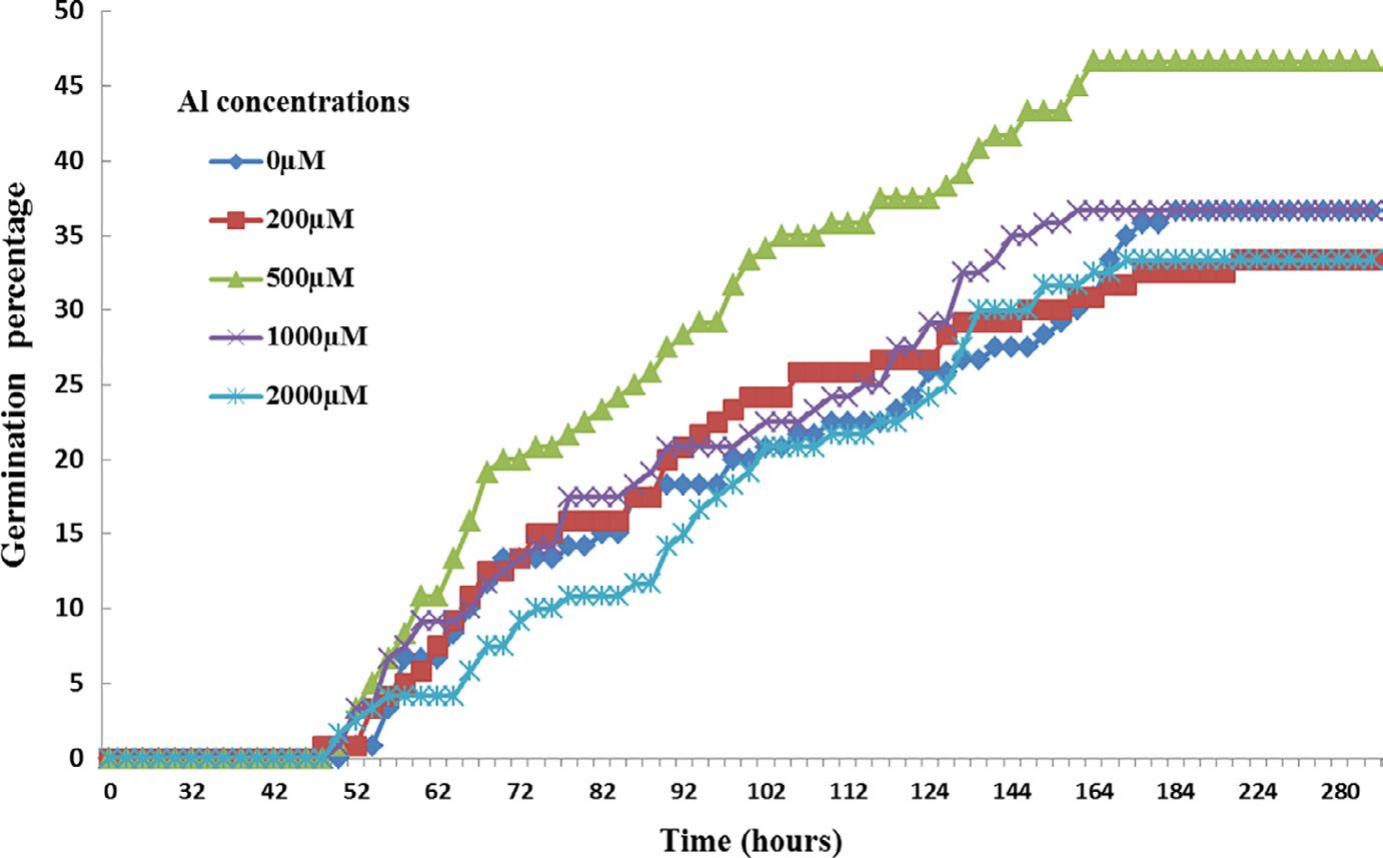
Effect of increasing doses of Al (0, 200, 500, 1,000, and 2,000 μM) on the germination percentage (GP) in Cucumis sativus seeds

Our results show that the best percentage of germination (46.7%) is observed with a dose of 500 μM of Al compared to the control where the germination percentage is equal to 36.7%. However, the addition of Al 200 and 2,000 μM slightly inhibited this physiological process. In fact, the percentage of germination did not exceed 33.3% (Figure 1).

As for the other parameters (Table 1), it was reported that the shortest T50 (80 hr) is recorded for 200 μM, Al. Indeed, this parameter is inversely correlated with the percentage of germination but the variations were not significant at *p* < .05. From the other side, the best germination capacity was observed with a concentration of 500 μM, Al. However, a dose of 2,000 μM Al decreases this parameter. Nevertheless, these variations in germination capacity were not significant at *p* < .05.

**TABLE 1:** Variation of germination parameters (T_50_, GCp, GVC, GI) under the effect of increasing doses of Al (0, 200, 500, 1,000, and 2,000 µM)

	0 µM	200 µM	500 µM	1,000 µM	2,000 µM
T50	89.25 ± 8.2^a^	81.75 ± 6.3^a^	91.25 ± 6.8^a^	91.25 ± 6.8^a^	98.75 ± 6.3^a^
GCp	0.21 ± 0.04^a^	0.24 ± 0.04^a^	0.25 ± 0.03^a^	0.23 ± 0.03^a^	0.17 ± 0.01^a^
GVC	56.85 ± 2.8^a^	64.11 ± 3.7^a^	62.66 ± 2.2^a^	62.53 ± 2.3^a^	57.74 ± 2.3^a^
GI	5.81 ± 0.5^a^	5.70 ± 1.03^a^	6.60 ± 1.1^a^	6.75 ± 0.9^a^	5.80 ± 0.6^a^

Note

Data are mean values ± *SE*, *n* = 8. Different letters are significantly different at *p* < .05.

Our results show that the increase in Al concentration does not cause any significant variation in the germination velocity coefficient at *p* < .05. Yet, there was a low increase in this coefficient compared to the control (0 μM, Al). For the germination index (GI), a slight stimulation under the effect of 500 and 1,000 μM of Al occurred. This behavior reminded us of the percentage of germination. However, the variations of this parameter are not significant at *p* < .05 (Table 1).

### 3.2 Biomass production and water content

**FIGURE 2:**
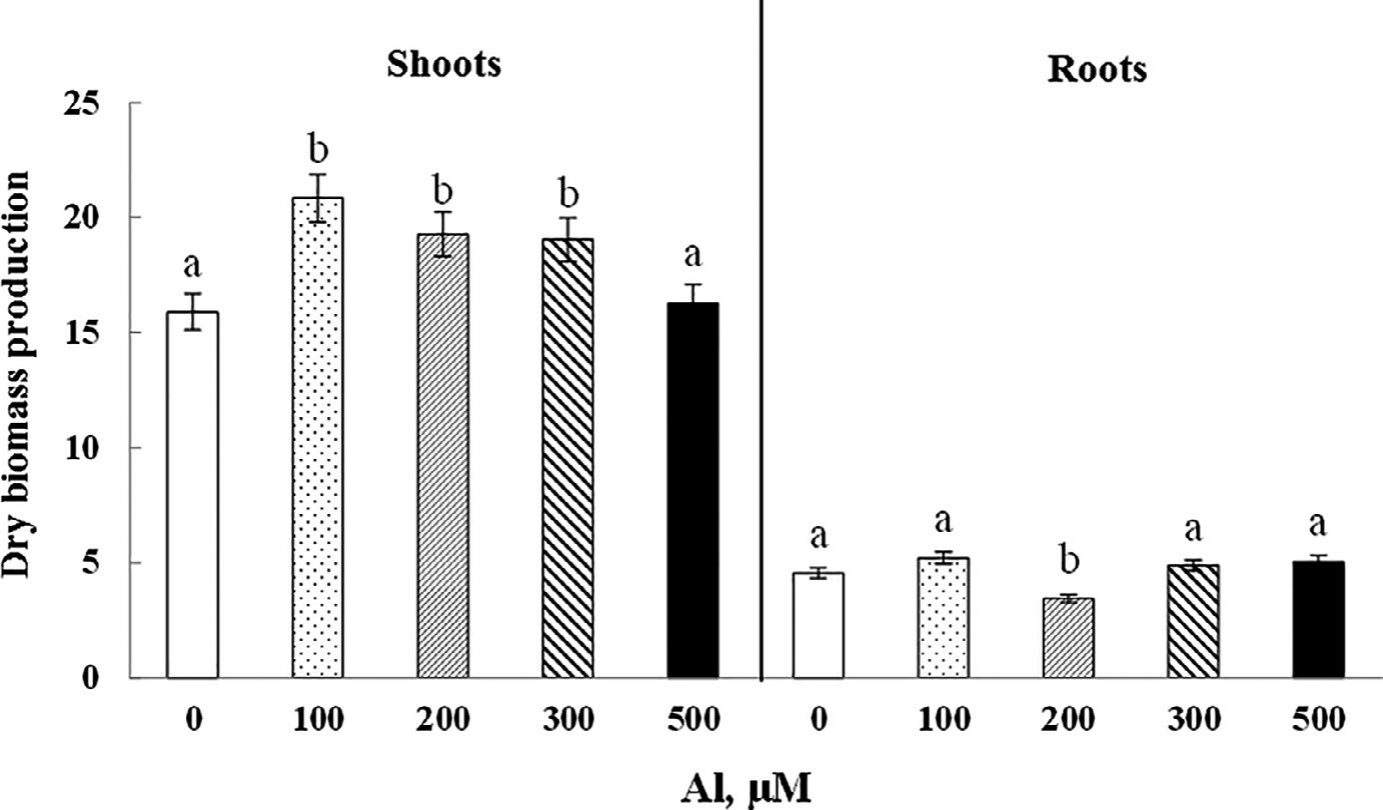
Variations of dry biomass production in roots and shoots of Cucumis sativus treated with 0, 100, 200, 300, and 500 μM Al. Data are mean values ± *SE*, *n* = 10. Bars marked with different letters are significantly different at *p* < .05

Concerning the biomass production, a significant improvement in the shoots of cucumber plants was noticed. The growth stimulation reached 31.0%, 21.2%, and 19.8% with 100, 200, and 300 µM Al, respectively (Figure 2). A slight increase but not significant (*p* > .05), in the dry biomass production of the plant's roots treated with 100 µM Al was observed. However, the decrease of root growth is significant in plants treated with 200 µM Al (Figure 2).

**FIGURE 3:**
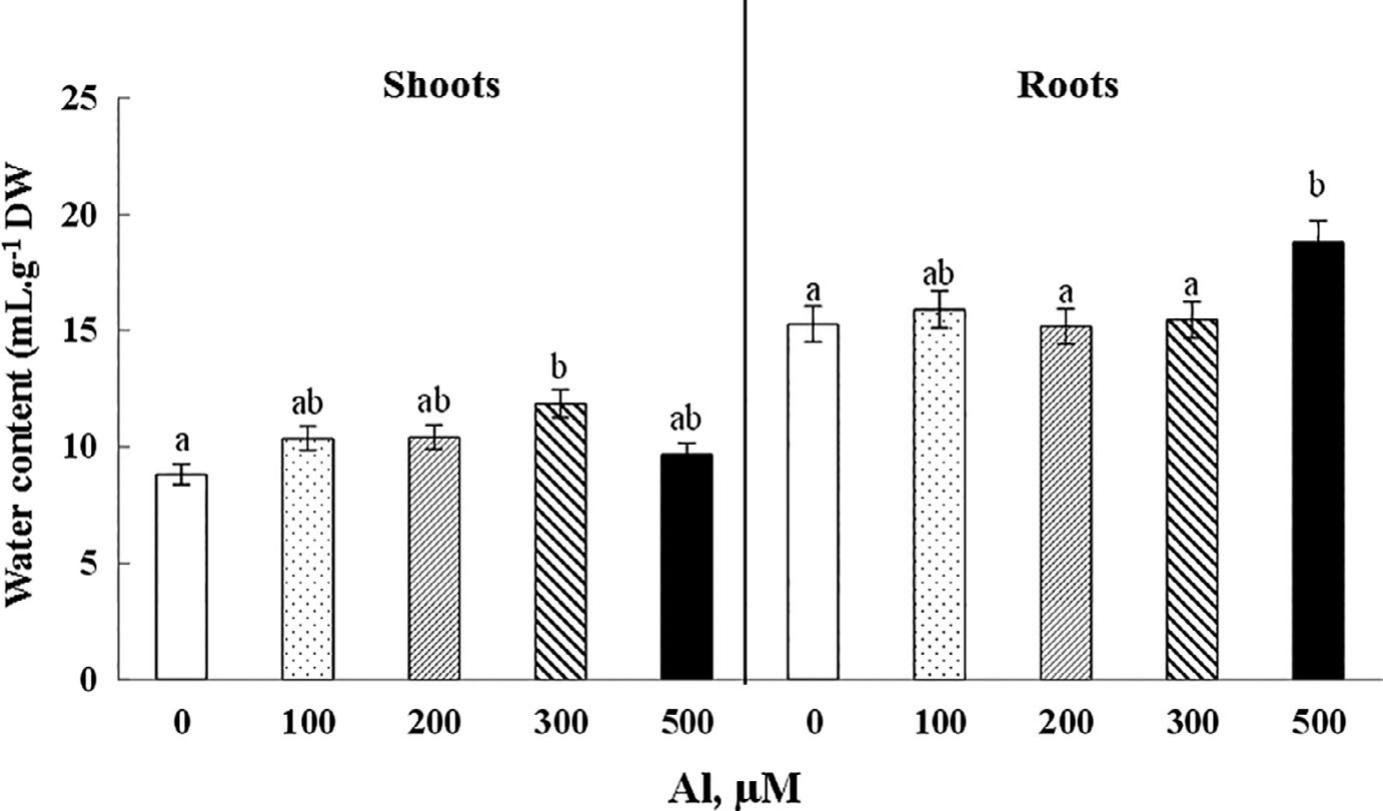
Variation of water content in roots and shoots of Cucumis sativus plants treated with 0, 100, 200, 300, and 500 µM Al. Data are mean values ± *SE*, *n* = 10. Bars marked with different letters are significantly different at *p* < .01

The water content of the aerial parts increased significantly (*p* < .1) with 34.5% in plants irrigated by 300 µM Al compared to the control plants. Similarly, water content in roots showed an increase of 22.9% in plants treated with 500 µM Al (*p* < .1) (Figure 3).

### 3.3 Aluminum content

**FIGURE 4:**
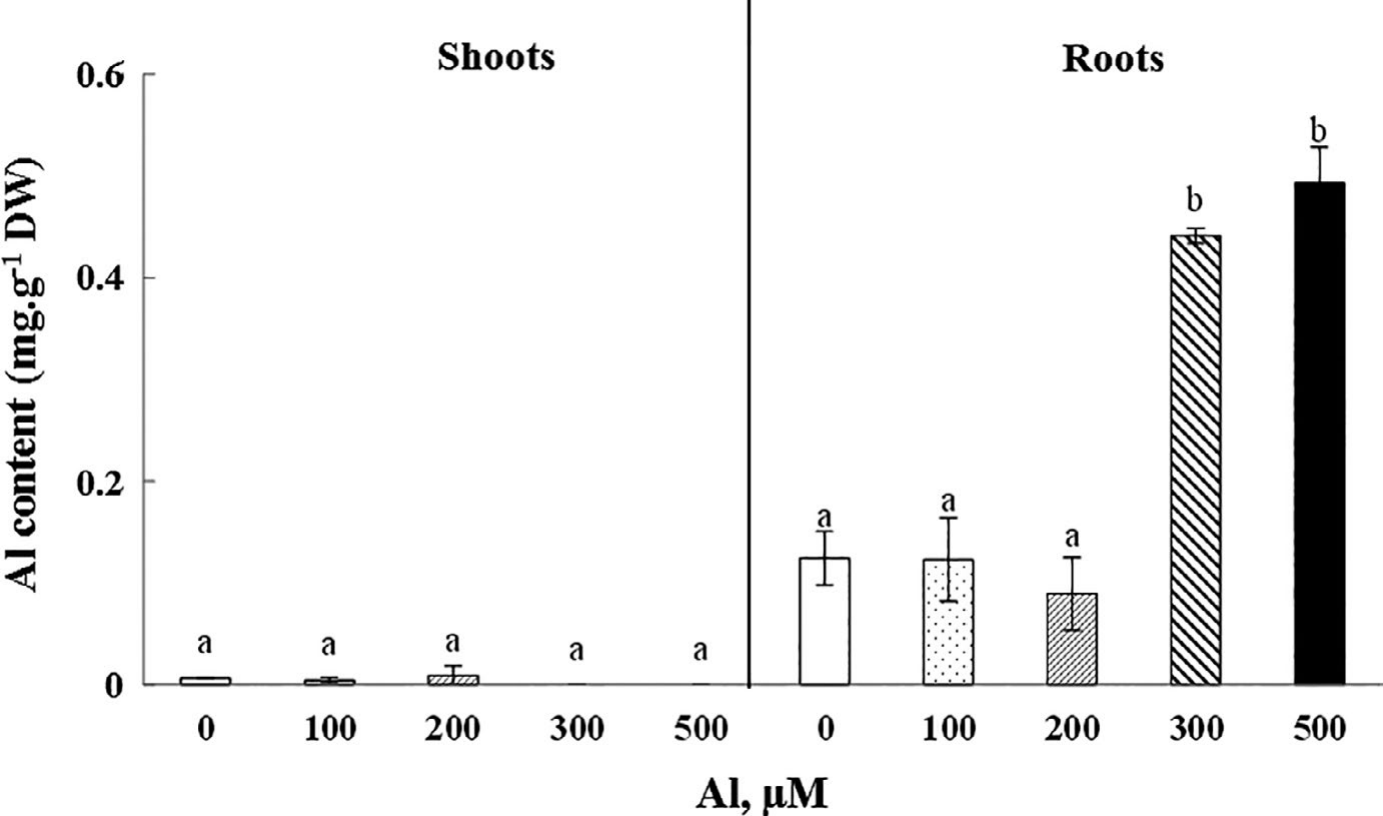
Variation of Al contents in roots and shoots of Cucumis sativus plants treated with 0, 100, 200, 300, and 500 µM Al. Data are mean values ± *SE*, *n* = 10. Bars marked with different letters are significantly different at *p* < .05

The accumulation of Al occurred mainly in the roots of cucumber plants, and less importantly in the shoots, which shows a high retention of this cation by the underground parts of cucumber plants. The Al contents increased significantly (*p* < .05) with the increasing of Al concentrations μM in irrigation solutions (Figure 4). The maximum accumulation reached 296.6% with 500 µM of Al when compared to the control.

### 3.4 Enzyme activities

**FIGURE 5:**
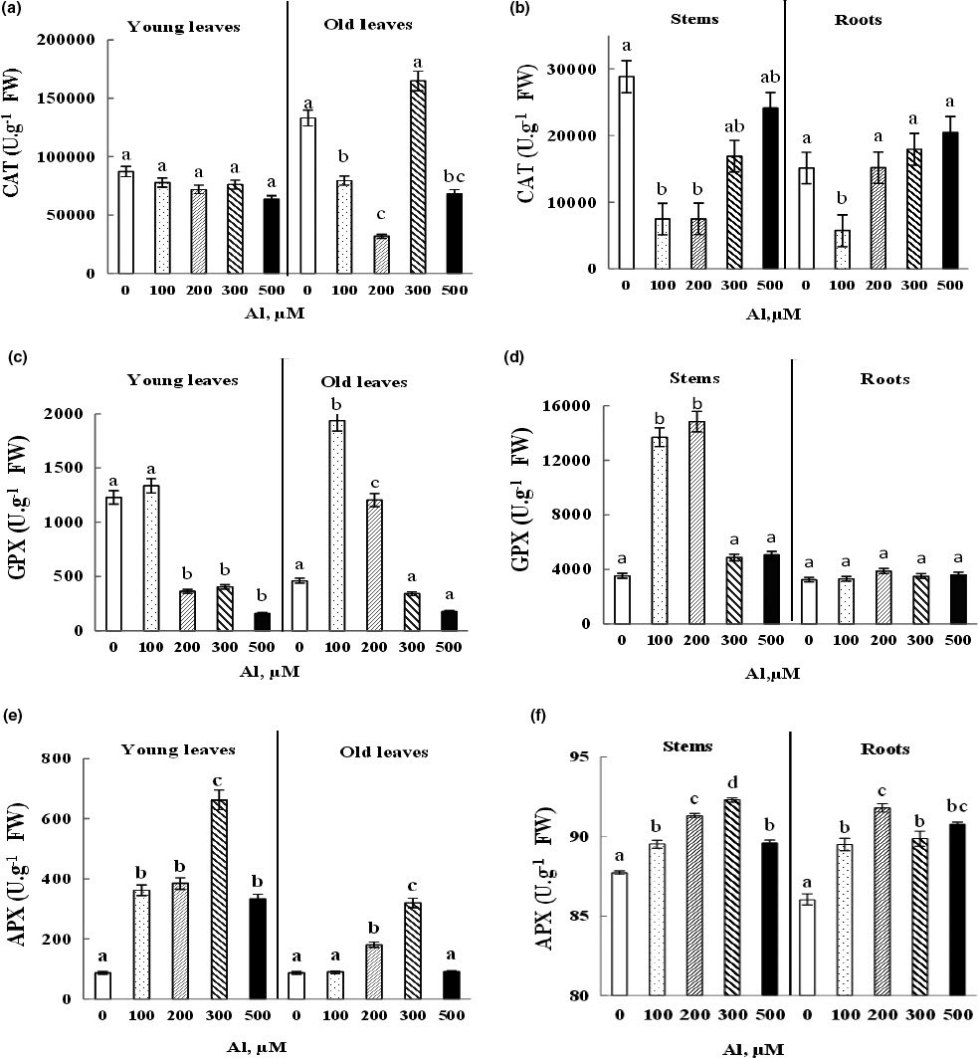
Variation of CAT, GPX and APX activities in young and old leaves, stems and roots of Cucumis sativus plants treated with 0, 100, 200, 300, and 500 µM Al. Data are mean values ± *SE*, *n* = 10. Bars marked with different letters are significantly different at *p* < .05

The variation of the enzymatic activities in the different organs of *Cucumis sativus* plants under the effect of the increasing doses (0, 100, 200, 300, and 500 μM) of Al is presented in Figure 5. Results showed that the addition of Al^3+^ to the irrigation solutions did not induce any significant variation (*p* < .05) in CAT activity in young leaves (Figure 5a). However, there was a significant inhibition in old leaves with 100, 200, and 500 μM Al and in the stems treated with 100 and 200 μM Al, yet in roots, this inhibition was observed only with a dose of 100 µM (Figure 5b).

Concerning the GPX activity, there was a significant decrease (*p* < .05) in the activity of this antioxidant enzyme in young leaves taken from plants treated with 200, 300, and 500 μM Al (Figure 5c). On the other hand, the old leaves and the stems of the plants treated with 100 and 200 μM Al show a clear and significant stimulation of GPX activity. For the roots, no variation of GPX was observed (Figure 5d).

It has been also reported that the Al‐induced stress induces a significant increase (*p* < .05) in APX activity in the various organs of cucumber plants. This improvement was higher in young leaves and old leaves of plants mainly with 300 µM of Al (Figure 5e). The enhancement of APX activity was also noticed in the stems and roots with a maximum stimulation at 300 and 200 µM, respectively (Figure 5f).

## 4 DISCUSSION

### 4.1 Germination

The germination process is controlled by several mechanisms. It is necessary for the growth and development of the embryo, resulting in the eventual production of a new plant. As a matter of fact, germination is regulated by genotypic characteristics but also by environmental conditions. Inappropriate conditions may compromise the ability of seeds to sprout. In fact, it has been proven that thermal stress and drought stress affected germination parameters in four chickpea varieties (Sleimi et al., 2013). Our study of germination carried out on cucumber seeds treated with increasing doses of Al shows that the best germination percentage was reached with 500 μM Al. These results are in agreement with those observed in *Cucurbita pepo* treated with different concentrations of copper, which shows an increase of 40% in germination percentage with 1,000 μM of Cu (Bankaji et al., 2017). Similarly, a slight stimulation of germination was observed in *Dorycnium pentaphyllum* with 10 μM of Cd (Lefevre et al., 2009). Actually, some plant species have developed the ability to tolerate the stress induced by metals. It is also necessary to consider the role played by the seed coat, which is a barrier between the embryo and the surrounding environment (Carlson et al., 1991).

The inhibitory effect of TME on germination observed essentially with 200 and 2,000 μM Al was also observed in other studies. For example, the results of Maheshwari and Dubey (2008) show that a dose of 400 μM Ni results in a decline of 12% in germination in *Oryza sativa*. Further, some authors explain the reduction in seed germination in several plant species treated by Al mainly by hormonal imbalance (Roshani et al., 2014).

### 4.2 Growth

Plants show different behaviors as a response to abiotic stresses like metallic stress, salinity, temperature, and drought. Some plants are able to tolerate these conditions, others are negatively affected. For example, the increase in temperature apparently had a positive effect in plant biomass which was promoted by the rising temperature by harvesting more carbon from the atmosphere (Caçador et al., 2016). Moreover, the growth of *Sesuvium portulacastrum* decreased significantly at high salinity levels (600 to 1,000 mM) (Messeddi et al., 2001). As for Al stress, the biomass production is generally limited in plants treated with Al (Inostroza‐Blancheteau et al., 2012).

This effect was also emphasized with the results found in this study; Al caused a slight significant decrease in root biomass dry with 200 μM. Similarly, previous studies have also shown the inhibitory effect of Al on growth in two cucurbitaceae subjected to Al stress; in cucumber (Rouphael et al., 2016) and in zucchini (Rouphael et al., 2015). In fact, the lessening of synthesis and transport of auxin under the effect of Al toxicity (Wang et al., 2016) seems to be the main reason of the root growth alteration, since it is an essential phytohormone for rhizogenesis favoring the roots growth and development.

On the other hand, low doses of Al resulted in a slight stimulation of growth in shoots. This phenomenon has frequently been noticed, though Al does not being regarded as an essential nutrient. Actually, Al application gives rise to an increase of chaperone proteins in plants, inducing an improvement of the tolerance to adverse environmental conditions through proper maintenance of proteins and cellular homeostasis, as reported in citrus leaves (Li et al., 2016) and in soybean (Zhen et al., 2007). Aluminum did not have any negative impact on water content; in fact, it even showed a slight increase. Unlike our results, authors explained the hydration deficit resulting from the low efficiency of water absorption by the involvement of aquaporin family members in Al transport (Wang et al., 2017).

### 4.3 Aluminum accumulation

In plants, metallic ions may have a different distribution rate, so some TME are immobilized and accumulated in root tissues such as Cd (Labidi et al., 2021), Fe (Roshani et al., 2014), As (Raab et al., 2007) and Al (Fan et al., 2020). Other cations, like Ba, are more freely transported to the aerial parts (Sleimi et al., 2021). Actually, TME can be translocated differently within the plant (Raab et al., 2007). The rates of TME in plants are distributed according to the accumulation gradient: roots > stems > leaves > seeds > fruits. When taken from the plant, metallic ions attach largely to the cell walls; which may explain their concentration in the roots, as for example, zinc (Lasat et al., 2000).

Results described in this work indicate that Al is accumulated in different organs of *Cucumis sativus* plants. There was a strong accumulation of Al in roots (0.49 mg g^−1^ DW), however, in the aerial parts of plants treated with 500 μM, the Al contents are 4 times lower, about 0.11 mg g^−1^ DW. Likewise, the accumulation of metal ions in the root was also noted in *Suaeda fructicosa* and *Atrriplex halimus* treated with Cd^2+^, Cu^2+^, Pb^2+^, and Zn^2+^, where the endogenous concentrations of these elements increased mainly in roots, depending on the increase in the concentration of these TME used in treatment (Bankaji et al., 2019). Actually, this can be explained by the low mobility of TMEs from the roots to the aerial parts and the immobilization of these elements in the roots (Martins & Mourato, 2006).

According to Jansen et al., (2002), the Al‐hyperaccumulator plants are able to retain more than 1.0 mg g^−1^ in the aerial parts. Therefore, we cannot consider the species *Cucumis sativus* as a hyperaccumulator plant of Al since the accumulation occurs mostly in the roots and Al contents in shoots do not exceed the threshold.

### 4.4 Antioxidant enzymes

Indeed, like the other abiotic constraints, metallic stress can generate a state of oxidative stress that is characterized by the appearance, accumulation, and production of ROS such as singlet oxygen, superoxide anion, hydroxyl radical, and hydrogen peroxide (Anjum et al., 2016).

Plants are able to neutralize these toxic forms by implementing several physiological and metabolic mechanisms and also anatomical and morphological adaptations (Steffens, 2014). Among these mechanisms, we can mention the enzymatic antioxidant defense system. Moreover, excessive doses of TME may result in inhibition or stimulation of the activity of antioxidant enzymes such as CAT, GPX, and APX which play a crucial role in the neutralization of ROS (Zheng et al., 2010).

In fact, several authors reported a decrease in CAT activity in response to metallic stress such as in *Brassica juncea* treated with 200, 300, and 500 µM of Ba (Bouslimi et al., 2021). Similarly, CAT activity decreased in *Atriplex halimus* treated with 400 μM of Cu (Bankaji et al., 2016). Likewise, in this work, there was a decline in CAT activity that was noticed especially in old leaves and stems. This decrease can be explained by the association of this enzyme with peroxisome proteases or with photoinactivation (Sandalio et al., 2001). Unlike our data, Arundhathi et al., (2016) trials showed that under the effect of 6 mM of Al, there was a considerable and significant increase in CAT activity (approximately 190%) in *Vigna trilobata* (L.). Moreover, *Triticum aestivium* treated with 500 μM Pb presented stimulation in CAT activity (Kaur et al., 2013). Yang and Poovaiah (2002) suggest that the increase in CAT activity is related to the increase in the intracellular concentration of H_2_O_2_ and Ca.

As for GPX, it is localized in extracellular spaces, the cell wall, the cytosol, and the vacuole, and plays a crucial role in antioxidant defense by consuming H_2_O_2_. It has a low affinity for aromatic electrons (Asada, 1999). In addition, GPX is sensitive to TME within the cell. These elements are able to modify its activity, for example, the activity of GPX was negatively affected in *Suaeda fruticosa* exposed to cadmium stress (Bankaji et al., 2015). Our results showed that in young leaves, Al was able to induce a decrease in GPX activity. Equally this decrease was reported in Pea roots treated with Cd (Głowacka et al., 2019). Moreover, our trials revealed that the dose of 100 or 200 μM was able to stimulate the activity of this enzyme in the stems and old leaves of *Cucumis sativus* plants. The same results were found in *Vigna trilobata* (L.) Verde where there was a 31% increase under the effect 6 mM of Al (Arundhathi et al., 2016). This is also consistent with the results found in *Helianthus* *annus* L. (Jouili et al., 2011) under the effect of Al‐induced stress where there was an increase in GPX activity.

The enzyme APX has a crucial role in neutralizing ROS during stress by reducing H_2_O_2_ to H_2_O using ascorbic acid as an electron donor (Gill & Tuteja, 2010). According to our results, the APX activity was stimulated by 300 μM Al in the young and old leaves and by 200 μM Al in the stems and roots. This increase has been reported also in *Triticum aestivium* due to cadmium stress (Khan et al., 2007). Also, there was a significant increase in the transcript levels of all APX encoding genes in rice after 8 hr of exposure to 20 ppm of Al (Rosa et al., 2010).

## 5 CONCLUSION

Considering all of the above, metallic stress induced by Al might improve germination percentage. On the other hand, Al has a positive effect on the growth of plants, and its accumulation occurs mainly in the root. These results offer encouraging prospects for assessing the accumulation of TME at the fruiting stage and following the food quality of the fruit.

## CONFLICT OF INTEREST

The authors have declared that no conflicts of interests exist.
